# Supplementary material for: Annotation-free multi-organ anomaly detection in abdominal CT using free-text radiology reports: a multi-centre retrospective study
Source: eBioMedicine. 2024 Nov 28;110:105463. doi: 10.1016/j.ebiom.2024.105463 (PMC11663761; doi:10.1016/j.ebiom.2024.105463)
Supplement: Supplementary Figures and Tables [file mmc1.pdf]

Contents of Supplementary Information

Supplementary Figure 1 – page 2

Supplementary Figure 2 – page 3

Supplementary Table 1 – page 4,5

Supplementary Table 2 – page 6,7

Supplementary Table 3 – page 8

Supplementary Table 4 – page 9

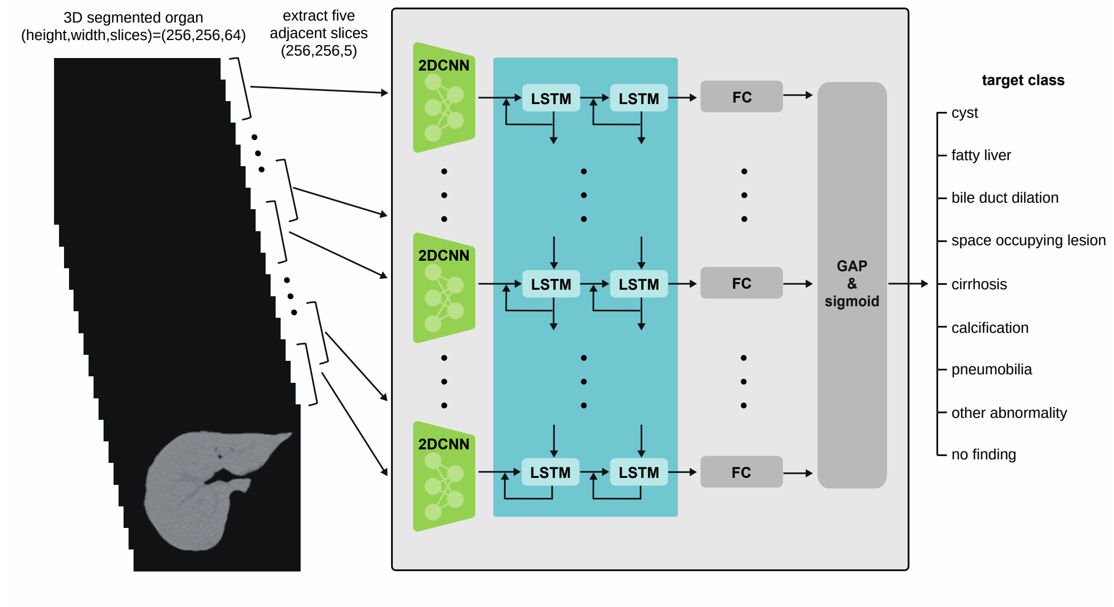

Supplementary Figure 1: Outline of the anomaly detection module.

The figure shows a schematic diagram of our deep learning model for detecting abnormal findings. Our model employs a multiple instance learning model to integrate different slice images. Five adjacent slices of segmented organ images are sequentially extracted with an overlap of two slices and input into a 2D convolutional neural network (CNN). Outputs from the CNN are fed into a Long Short-Term Memory (LSTM) network to share information between slices. Fully connected layers (FC) then act as a classifier. Information across slices is aggregated using global average pooling (GAP), followed by a sigmoid activation function and a cross-entropy loss function. During training, the model is trained as a multi-class classifier including no findings and multiple abnormal findings. During inference, it simplifies to binary classification, detecting only the presence or absence of anomalies.

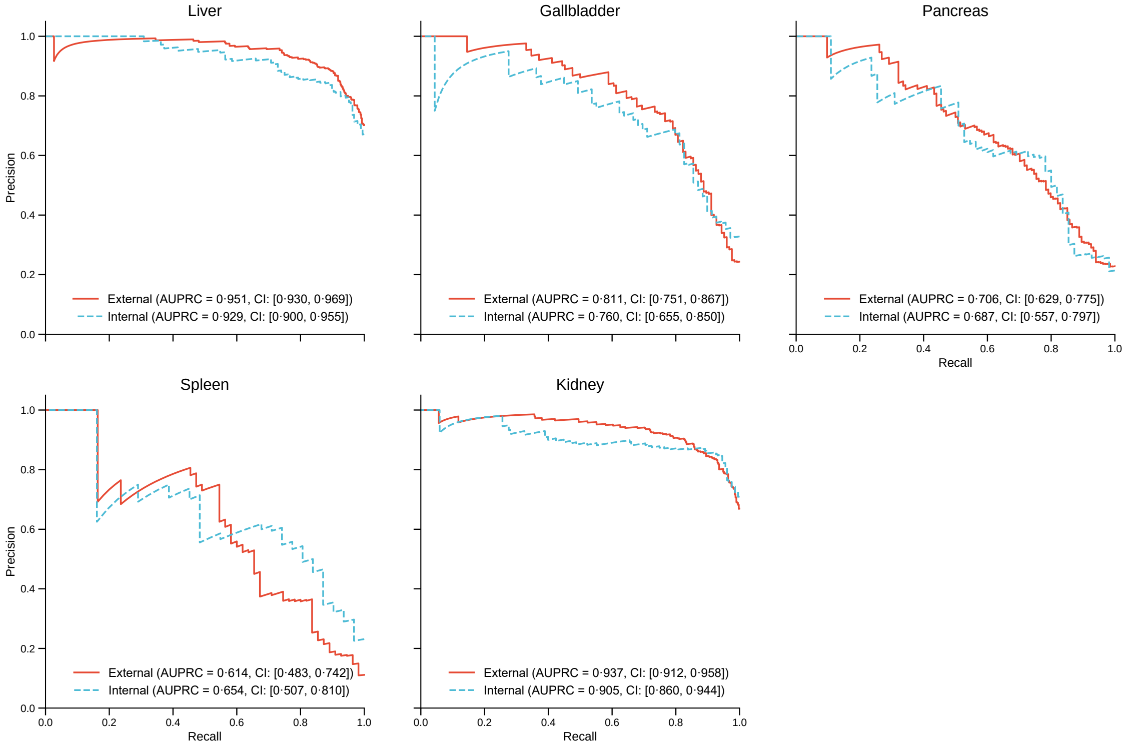

Supplementary Figure 2: Precision-recall curves for the anomaly detection models

The precision-recall curve for each organ in the internal and external test cohort. The curve presents the precision and recall across different cutoffs. The values on each graph are the areas under the precision-recall curve (AUPRCs) and their 95% confidence intervals for each cohort.

Supplementary Table 1: Images per vendor in the internal training cohort

	Manufacturer	Model Name	Keio	Okayama	Tokyo
**Liver**	Canon Medical Systems	Aquilion ONE	273	2702	1300
		Aquilion Precision		2527	2336
		Aquilion PRIME			8905
		Aquilion Prime SP			1858
	GE Healthcare	Discovery CT750 HD	4256	2678	5318
		Revolution CT	3190		8164
		Revolution EVO	1063		
		BrightSpeed	601		
	Siemens	SOMATOM Definition		2568	
		SOMATOM go.Top		1030	
		NAEOTOM Alpha		79	
**Gallbladder**	Canon Medical Systems	Aquilion ONE	54	1064	1094
		Aquilion Precision		902	1986
		Aquilion PRIME			7576
		Aquilion Prime SP			1524
	GE Healthcare	Discovery CT750 HD	646	1128	4739
		Revolution CT	540		6957
		Revolution EVO	264		
		BrightSpeed	37		
	Siemens	SOMATOM Definition		978	
		SOMATOM go.Top		412	
		NAEOTOM Alpha		41	
**Pancreas**	Canon Medical Systems	Aquilion ONE	105	1235	1399
		Aquilion Precision		1171	2508
		Aquilion PRIME			9290
		Aquilion Prime SP			1867
	GE Healthcare	Discovery CT750 HD	1045	1315	5942
		Revolution CT	783		8418
		Revolution EVO	297		
		BrightSpeed	39		
	Siemens	SOMATOM Definition		1166	
		SOMATOM go.Top		414	
		NAEOTOM Alpha		46	
**Spleen**	Canon Medical Systems	Aquilion ONE	39	901	1326
		Aquilion Precision		817	2304
		Aquilion PRIME			8645
		Aquilion Prime SP			1756
	GE Healthcare	Discovery CT750 HD	446	971	5569
		Revolution CT	352		7904
		Revolution EVO	199		
		BrightSpeed	22		
	Siemens	SOMATOM Definition		865	
		SOMATOM go.Top		315	
		NAEOTOM Alpha		34	

**Kidney**	Canon Medical Systems	Aquilion ONE	411	4516	2407
		Aquilion Precision		4261	4638
		Aquilion PRIME			17102
		Aquilion Prime SP			3225
	GE Healthcare	Discovery CT750 HD	6451	4798	10437
		Revolution CT	4997		15018
		Revolution EVO	1788		
		BrightSpeed	441		
	Siemens	SOMATOM Definition		4488	
		SOMATOM go.Top		1734	
		NAEOTOM Alpha		148	
		Unknown		4	

Data are n.

Supplementary Table 2: Images per vendor in the internal and external test cohorts

	Manufacturer	Model Name	Tokyo	Keio	Okayama	Ehime	Juntendo	Kyoto	Kyushu	Osaka	Tokushima
**Liver**	Canon Medical Systems	Aquilion ONE	15	3	30	29	89	41	58	39	100
		Aquilion Precision			16				19	29	
		Aquilion PRIME	10					59	1		
		Aquilion Prime SP	34								
	GE Healthcare	Discovery CT750 HD	13	44	24						
		Revolution CT	28	36						32	
		Revolution EVO		12							
		BrightSpeed		5							
	Siemens	SOMATOM go.Top			9						
		SOMATOM Force				45					
		NAEOTOM Alpha			21						
	Philips	iCT 256				26	11				
		IQon - Spectral CT							22		
**Gallbladder**	Canon Medical Systems	Aquilion ONE	13	2	25	23	85	40	48	33	92
		Aquilion Precision			14				15	24	
		Aquilion PRIME	9					50			
		Aquilion Prime SP	30								
	GE Healthcare	Discovery CT750 HD	12	41	22						
		Revolution CT	27	30						28	
		Revolution EVO		9							
		BrightSpeed		5							
	Siemens	SOMATOM go.Top			8						
		SOMATOM Force				40					
		NAEOTOM Alpha			20						
	Philips	iCT 256				25	11				
		IQon - Spectral CT							19		
**Pancreas**	Canon Medical Systems	Aquilion ONE	15	3	30	29	89	41	58	39	100
		Aquilion Precision			15				19	29	
		Aquilion PRIME	10					59	1		
		Aquilion Prime SP	34								
	GE Healthcare	Discovery CT750 HD	13	44	24						
		Revolution CT	28	36						32	

		Revolution EVO		12							
		BrightSpeed		5							
	Siemens	SOMATOM go.Top			9						
		SOMATOM Force				45					
		NAEOTOM Alpha			21						
	Philips	iCT 256				26	11				
		IQon - Spectral CT							22		
**Spleen**	Canon Medical Systems	Aquilion ONE	15	3	30	29	89	40	58	35	100
		Aquilion Precision			15				19	29	
		Aquilion PRIME	10					58			
		Aquilion Prime SP	34								
	GE Healthcare	Discovery CT750 HD	13	42	24						
		Revolution CT	28	35						31	
		Revolution EVO		12							
		BrightSpeed		5							
	Siemens	SOMATOM go.Top			9						
		SOMATOM Force				45					
		NAEOTOM Alpha			21						
	Philips	iCT 256				26	11				
		IQon - Spectral CT							22		
**Kidney**	Canon Medical Systems	Aquilion ONE	30	6	60	56	176	81	113	76	198
		Aquilion Precision			31				38	57	
		Aquilion PRIME	20					117	2		
		Aquilion Prime SP	67								
	GE Healthcare	Discovery CT750 HD	26	86	47						
		Revolution CT	55	72						62	
		Revolution EVO		24							
		BrightSpeed		10							
	Siemens	SOMATOM go.Top			18						
		SOMATOM Force				88					
		NAEOTOM Alpha			42						
	Philips	iCT 256				49	21				
		IQon - Spectral CT							44		

Data are n.

Supplementary Table 3: Performance of the multi-organ segmentation model

	Liver	Spleen	Pancreas	Gallbladder	Left kidney	Right kidney
**DSC**	0.981 (0.974–0.986)	0.979 (0.972–0.985)	0.911 (0.882–0.934)	0.937 (0.913–0.957)	0.973 (0.963–0.980)	0.972 (0.965–0.979)
**NSD**	0.871 (0.820–0.937)	0.934 (0.902–0.965)	0.77 (0.706–0.831)	0.865 (0.784–0.935)	0.918 (0.887–0.952)	0.923 (0.893–0.950)

Data are median (IQR). DSC=dice similarity coefficient. NSD=normalised surface dice.

Supplementary Table 4: Disease extraction performance per institution by the information extraction schema.

	Metrics	Tokyo	Keio	Okayama	Ehime	Juntendo	Kyoto	Kyushu	Osaka	Tokushima
**Liver**	accuracy	94.7	96.6	94.7	98.6	98.7	98.4	92.8	97.6	98.6
	sensitivity	91.9	66.7	100.0	90.0	100.0	50.0	100.0	100.0	94.4
	specificity	97.4	100.0	93.5	100.0	98.5	100.0	88.4	96.9	100.0
	f1	94.4	80.0	88.0	94.7	96.3	66.7	91.2	95.2	97.1
**Gallbladder**	accuracy	98.5	85.7	93.9	92.3	100.0	100.0	96.8	100.0	100.0
	sensitivity	98.1	100.0	91.3	91.7	100.0	100.0	100.0	100.0	100.0
	specificity	100.0	83.3	100.0	92.9	100.0	100.0	77.8	100.0	100.0
	f1	99.1	66.7	95.5	91.7	100.0	100.0	98.2	100.0	100.0
**Pancreas**	accuracy	96.4	90.9	95.0	83.7	97.2	90.9	93.6	95.3	88.6
	sensitivity	98.6	100.0	93.1	100.0	100.0	100.0	96.5	96.7	90.9
	specificity	83.3	87.5	100.0	75.9	83.3	87.5	85.7	92.0	87.5
	f1	97.9	85.7	96.4	80.0	98.4	85.7	95.7	96.7	83.3
**Spleen**	accuracy	96.4	85.7	100.0	95.7	97.5	100.0	98.6	98.7	93.8
	sensitivity	97.4	100.0	100.0	93.3	97.0	100.0	98.5	98.5	100.0
	specificity	80.0	80.0	100.0	100.0	100.0	100.0	100.0	100.0	87.5
	f1	98.1	80.0	100.0	96.6	98.5	100.0	99.2	99.3	94.1
**Kidney**	accuracy	96.5	99.1	95.3	98.6	99.2	95.7	96.3	96.6	98.9
	sensitivity	98.8	83.3	100.0	100.0	100.0	100.0	98.9	100.0	100.0
	specificity	93.2	100.0	93.7	98.5	98.8	95.0	93.0	96.6	98.7
	f1	97.1	90.9	91.5	80.0	98.7	85.7	96.8	57.1	96.3

Data are %.
